# Longitudinal analysis of the enteric virome in paediatric subjects from the Free State Province, South Africa, reveals early gut colonisation and temporal dynamics

**DOI:** 10.1016/j.virusres.2024.199403

**Published:** 2024-06-01

**Authors:** Milton Tshidiso Mogotsi, Ayodeji Emmanuel Ogunbayo, Phillip Armand Bester, Hester Gertruida O'Neill, Martin Munene Nyaga

**Affiliations:** a Next Generation Sequencing Unit and Division of Virology, Faculty of Health Sciences, University of the Free State, Bloemfontein 9300, South Africa; b Division of Virology, School of Pathology, Faculty of Health Sciences, University of the Free State, Bloemfontein 9300, South Africa; c Department of Microbiology and Biochemistry, Faculty of Natural and Agricultural Sciences, University of the Free State, Bloemfontein 9300, South Africa

**Keywords:** Infant gut virome, Enteric viruses, *Caliciviridae*, *Picornaviridae*, *Sedoreoviridae*, Gastroenteritis, Metagenomics, Free state, South Africa

## Abstract

The gut of healthy neonates is devoid of viruses at birth, but rapidly becomes colonised by normal viral commensals that aid in important physiological functions like metabolism but can, in some instances, result in gastrointestinal illnesses. However, little is known about how this colonisation begins, its variability and factors shaping the gut virome composition. Thus, understanding the development, assembly, and progression of enteric viral communities over time is key. To explore early-life virome development, metagenomic sequencing was employed in faecal samples collected longitudinally from a cohort of 17 infants during their first six months of life. The gut virome analysis revealed a diverse and dynamic viral community, formed by a richness of different viruses infecting humans, non-human mammals, bacteria, and plants. Eukaryotic viruses were detected as early as one week of life, increasing in abundance and diversity over time. Most of the viruses detected are commonly associated with gastroenteritis and include members of the *Caliciviridae, Picornaviridae, Astroviridae, Adenoviridae*, and *Sedoreoviridae* families. The most common co-occurrences involved asymptomatic norovirus-parechovirus, norovirus-sapovirus, sapovirus-parechovirus, observed in at least 40 % of the samples. Majority of the plant-derived viruses detected in the infants’ gut were from the *Virgaviridae* family. This study demonstrates the first longitudinal characterisation of the gastrointestinal virome in infants, from birth up to 6 months of age, in sub-Saharan Africa. Overall, the findings from this study delineate the composition and variability of the healthy infants’ gut virome over time, which is a significant step towards understanding the dynamics and biogeography of viral communities in the infant gut.

## Highlights

• Gut microbial colonisation in infants is a crucial, yet intricate, process, characterised by minimal diversity at birth that increases with age.
• The infant gut virome is dominated by eukaryotic viruses, consisting primarily of human enteric viruses and non-human dietary viruses.
• The infants’ gut haboured high abundance of gastroenteritis-associated viruses, despite the absence of clinical symptoms.
• The presence of plant viruses in the gut of infants who are not exposed to solid food could suggest vertical transmission from mother to infant, or possibly, contact between infant and other family members.

## 1 Introduction

It is widely acknowledged that humans are hosts to a complex ecosystem consisting of a diverse community of bacteria, archaea, fungi, viruses, and other eukaryotes (Matijašić et al., 2020). This assemblage of microorganisms, referred to as the human microbiome, inhabits different anatomical compartments of the human body, with the gastrointestinal system being the most densely colonised. The human gut microbiome has a significant impact on both health and disease (Clemente et al., 2012; Lynch and Pedersen, 2016). Disturbances in the composition of gut microbes, termed dysbiosis, have been associated with illnesses like obesity (Arslan, 2014; Sankararaman et al., 2023), diabetes (Larsen et al., 2010; Zhu and Goodarzi, 2020), inflammatory bowel disease (IBD) (Mars et al., 2020), cancer (Yoshimoto et al., 2013), and neurological diseases (Socała et al., 2021). Although studies have established connections with IBD (Ansari et al., 2020), diabetes (Yang et al., 2021), liver infections (Lang et al., 2020), cancer (Nakatsu et al., 2018), and SARS-CoV-2 (Zuo et al., 2021), research on human gut viruses is still lagging behind. In addition, the normal commensal gut microbiome provides essential health benefits to its host. Studies have demonstrated the fundamental role of bacteriophages in the regulation of host innate and adaptive immunity, as well as its defence mechanisms (Barr et al., 2013; Popescu et al., 2021).

The gastrointestinal tract (GIT) is home to the most abundant viruses in the human body, collectively called the gut virome (Bushman and Liang, 2021). The gut virome is composed of diverse eukaryotic viruses capable of infecting humans and animals, prokaryotic viruses such as bacteriophages, and diet-derived plant viruses (Carding, Davis and Hoyles, 2017). While enteric viruses cause numerous infections in children, our understanding of the paediatric gut virome is limited. According to recent studies, the gut of healthy neonates is virus-free at birth, but within a week afterwards, colonisation by viruses and bacteria had already taken place (Liang and Bushman, 2021). Several reports have also shown that neonates host a great variety of eukaryotic viruses and bacteriophages (Lim et al., 2015; Liang, Zhao, et al., 2020; Mogotsi et al., 2020), with eukaryotic viruses increasing in abundance over time. Furthermore, metagenomic next generation sequencing (mNGS) techniques have revealed that the paediatric gut virome is dynamic, with high level of inter-personal variability, and some degree of intra-host stability over time (Kaelin et al., 2022).

Although factors shaping the human gut virome are well established, how these factors contribute to the gut virome structure and composition during the first few months of life, is not well understood. A number of studies have revealed a combination of factors, including diet, age, delivery mode, use of medication like antibiotics, geographic location, environment, socio-economic status, human behaviour like smoking guardians, and health status as main drivers of microbial colonisation of the infant gut (Milani et al., 2017; Gregory et al., 2020; Bushman and Liang, 2021). For instance, analysis of meconium samples from new-borns delivered vaginally revealed gut microbial communities similar to those in the mother's vagina, whereas microbes in the gut of neonates delivered via caesarean section (C-section) resembled those on the mother's skin (Dominguez-Bello et al., 2010). Likewise, comparison between breast-feeding infants and those on formula milk highlighted key differences in the gut microbial configurations, associated with the respective feeding types (Song, Dominguez-Bello and Knight, 2013).

Considering the significance of microbiome acquisition and the interplay between virome and microbiome on human health and disease, it is imperative to investigate the temporal evolution of the infant virome to understand factors that may impact health and disease states. The advent of mNGS techniques and bioinformatics tools have greatly facilitated studies of intestinal viruses and accelerated the discovery of new pathogens (Nooij et al., 2018; Noell and Kolls, 2019). In the current study, viral metagenomics, and bioinformatics was employed to characterise the gastrointestinal virome in longitudinally collected faecal samples from a cohort of infants in the Free State Province, South Africa. This cohort presents a great opportunity to understand the long-term evolution of the infants’ gastrointestinal virome during their first six months of life.

## 2 Results

### 2.1 Participant recruitment and faecal collection

A total of 52 study subjects were initially recruited for participation. However, only 17 of these were successfully sampled from birth to six months. Overall, 68 faecal samples were obtained from 17 infants, sampled four times over a period of approximately six months.

**Fig. 1:**
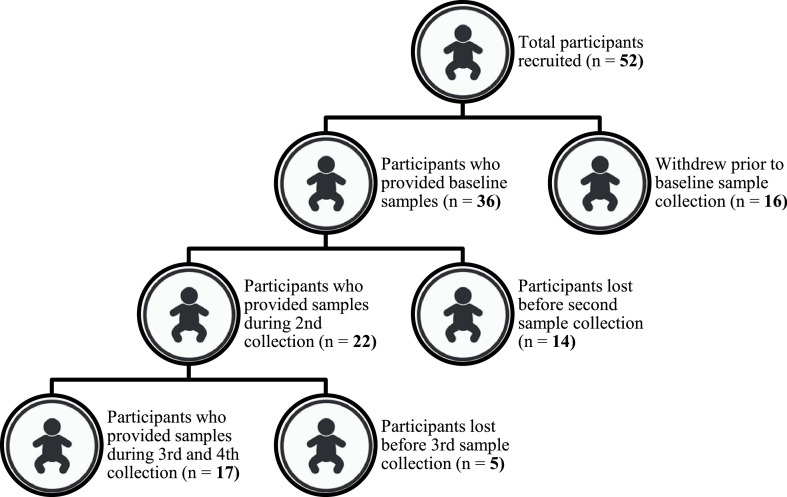
Flowchart of the study participant recruitment and longitudinal faecal sample collection. In total, 52 participants (mothers to new-borns and admitted pregnant women due to give birth) were recruited and enrolled into the study after giving consent. Of these, 16 withdrew prior to the start of sample collection due to reasons including cultural concerns, hesitancy, change of mind, and relocation to places outside the study area, while some could not be reached telephonically. With 36 participants remaining, a further 14 were lost to second follow-up, most of whom relocated. Lastly, five more participants were lost to third and fourth follow-ups as they could no longer be traced, while one infant had passed away. As a result, the study consisted of 17 participants, all of whom completed the cohort.

Recruitment and faecal sample collection procedure is illustrated in Fig. 1. In terms of demographics, the infants were born between May and August of 2021, of whom 70.6 % were females and 29.4 % males (Table 1). The mothers’ average gestational period was 38.5 weeks, and infants had an average birth weight of 2 875 gs. Fourteen of the infants were delivered vaginally, with three delivered via C-section.

**Table 1:** Subject characteristics and demographic information. (PRH = Pelonomi Regional Hospital; MUCPP = Mangaung University Community Partnership Programme; *F* = female; *M* = Male; HIV = Human Immunodeficiency Virus).

Infant Number	Infant ID	Sex	Hospital	Birth weight	Gestational period	Delivery route	HIV exposure	Diarrhoeal illness	Feeding type
**Infant 1**	***VRM1***	F	PRH	3790 g	39 weeks	Caesarean	No	No	Exclusive breast-feeding
**Infant 2**	***VRM2***	M	PRH	3140 g	38 weeks	Vaginal	No	No	Exclusive Breast-feeding
**Infant 3**	***VRM3***	F	MUCPP	2180 g	40 weeks	Vaginal	No	No	Exclusive Breast-feeding
**Infant 4**	***VRM4***	M	PRH	3210 g	38 weeks	Vaginal	No	No	Mixed feeding
**Infant 5**	***VRM5***	F	PRH	Unknown	39 weeks	Caesarean	Yes	No	Exclusive breast-feeding
**Infant 6**	***VRM6***	F	PRH	2300 g	34 weeks	Caesarean	No	No	Mixed feeding
**Infant 7**	***VRM7***	F	MUCPP	3100 g	41 weeks	Vaginal	Yes	No	Exclusive breast-feeding
**Infant 8**	***VRM8***	M	MUCPP	2700 g	39 weeks	Vaginal	Unknown	No	Exclusive breast-feeding
**Infant 9**	***VRM9***	F	PRH	3310 g	39 weeks	Vaginal	Yes	No	Exclusive breast-feeding
**Infant 10**	***VRM10***	F	PRH	3300 g	38 weeks	Vaginal	No	No	Exclusive breast-feeding
**Infant 11**	***VRM11***	M	PRH	2710 g	39 weeks	Vaginal	No	No	Exclusive breast-feeding
**Infant 12**	***VRM12***	F	PRH	3450 g	40 weeks	Vaginal	No	No	Exclusive breast-feeding
**Infant 13**	***VRM13***	F	PRH	2810 g	38 weeks	Vaginal	Yes	No	Exclusive breast-feeding
**Infant 14**	***VRM14***	F	PRH	3330 g	36 weeks	Vaginal	No	No	Exclusive breast-feeding
**Infant 15**	***VRM15***	F	PRH	2900 g	40 weeks	Vaginal	Yes	No	Exclusive breast-feeding
**Infant 16**	***VRM16***	F	PRH	3030 g	38 weeks	Vaginal	No	No	Exclusive breast-feeding
**Infant 17**	***VRM17***	M	PRH	3950 g	40 weeks	Vaginal	Yes	No	Exclusive breast-feeding

### 2.2 Clinical information

Clinically, *n* = 6 (35.3 %) of the infants were exposed to HIV upon birth and were all on highly active antiretroviral therapy (HAART). Regarding the health status of the study subjects, none of the infants had diarrhoea or gastrointestinal symptoms during sample collection. All except two were breast fed exclusively for the duration of the study, while the other two were on both breast and formula milk. Critical to this study was the vaccination status of the subjects which plays a key role in human virome composition and dynamics. Immunisation statuses of the infants were recorded during sample collection. At birth, the infants received Bacillus Calmette–Guérin (BCG) vaccine and oral polio vaccine (OPV), for protection against tuberculosis and poliovirus, respectively. At 6 weeks, the second dose of OPV was administered concurrently with oral drops of rotavirus vaccine, and a hexavalent combination vaccine, diphtheria-tetanus-acellular, pertussis-injectable, polio-Haemophilus influenza, b-Hepatitis B vaccine (DTaP-IPV-Hib-HBV), and pneumococcal conjugate vaccine (PCV). A second dose of DTaP-IPV-Hib-HBV was administered at 10 weeks, with a third dose at 14 weeks together with rotavirus vaccine and PCV. Lastly, at six months, all infants received the measles vaccine.

### 2.3 Metagenomic data overview

Metagenomic sequencing for infant gut virome profiling yielded a total of 28 593 948 reads, with an average of 420 499 reads per sample, ranging from 103 449 to 1 125 971 sequence reads (Table 2). Majority of the total reads (62.43 %) were assigned to bacteria, while 8.58 % of the total raw reads were assigned to viruses, most of which could be classified into family, genus, and species. The remainder of the classified reads included host genomes (not presented in the results).

**Table 2:** Overview of generated sequencing reads generated by mNGS.

Category	No. of raw reads	Classified reads	Unclassified reads	Bacterial reads	Viral reads	Other (i.e., host, etc.)
**Reads count**	28 593 948	22 684 259	5 909 689	17 852 090	2 453 206	2 378 963
**Percentage/ total raw reads**		79,33 %	20,67 %	62,43 %	8,58 %	8,32 %

### 2.4 Infant gut virome composition

In contrast to most viral metagenomics studies, the classified viral reads were dominated by eukaryotic viruses, with a total of 2 341 404 (95.4 %) reads, while 60 143 (2.5 %) reads were classified as bacteriophages, and the other 2.1 % assigned to viruses infecting other microorganisms such as fungi.

#### 2.4.1 Eukaryotic viruses

**Fig. 2:**
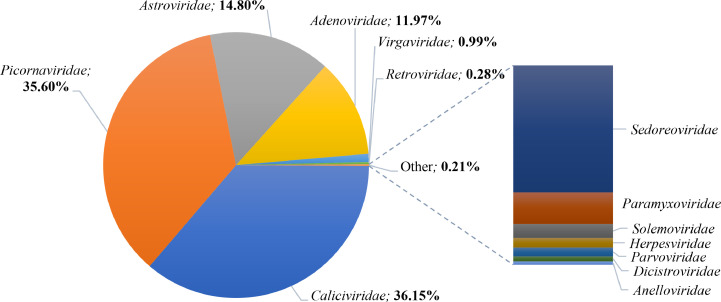
Taxonomic distribution of eukaryotic virus-related reads at family level.

More than two million reads were assigned to eukaryotic viruses, which were classified into 13 viral families of vertebrate- (10), invertebrate- (1), and plant-infecting viruses (2). *Caliciviridae* and *Picornaviridae* were the most abundant viral families, accounting for nearly 70 % of all eukaryotic viral reads. The remaining reads were further attributed to *Astroviridae* (14.8 %) and *Adenoviridae* (∼12.0 %) families (Fig. 2; Table S2). Other viral families identified included, in decreasing order of abundance, *Virgaviridae, Retroviridae, Sedoreoviridae, Paramyxoviridae, Solemoviridae, Herpesviridae, Parvoviridae, Dicistroviridae,* and *Anelloviridae* (Fig. 2; Table S2).

**Fig. 3:**
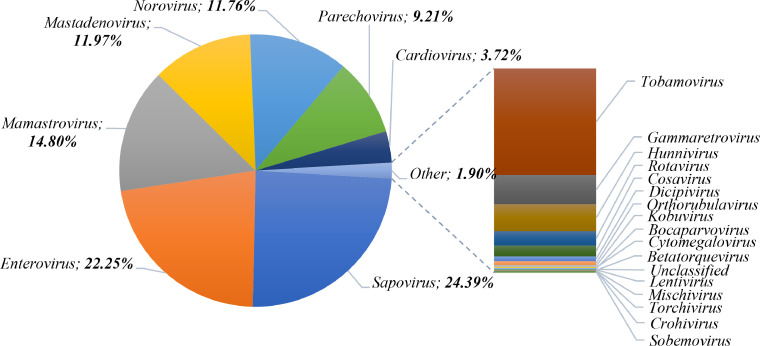
Proportion of the different viral genera detected in infants’ faecal samples.

At genus level, the eukaryotic viral reads could be classified into 23 viral genera, seven of which accounted for nearly 98 % of the reads (Fig. 3; Table S2). As anticipated, all seven genera include viruses mainly associated with gastroenteritis in infants. The genus *Sapovirus*, under *Caliciviridae* family, was the most predominant, constituting nearly a fourth of the reads. Equally abundant was the large genus of *Enterovirus*, which comprises numerous mammalian-infecting viruses, within the *Picornaviridae* family. Furthermore, *Mamastrovirus, Mastadenovirus, Norovirus, Parechovirus*, and *Cardiovirus* genera together represented nearly 50 % of the reads, while less than 2 % of the reads were distributed among several genera detected at relatively low abundance, including *Rotavirus, Cosavirus*, and *Aichivirus* (Fig. 3; Table S2). Additionally, *Tobamovirus*, one of the two detected genera of plant origin, was present in relatively significant numbers (23 131 reads).

Analysis of the metagenomic data revealed a total of 45 eukaryotic viral species in this study, of which 75 % possessed a positive-sense single-stranded RNA (ssRNA(+)) genome organisation (Table S2). These included mainly mammalian viruses in the families of *Picornaviridae, Caliciviridae*, and *Astroviridae*, five species of plant viruses in the *Virgaviridae* and *Solemoviridae* families, and one insect-infecting virus from the *Dicistroviridae* family. Human orthorubulavirus 4, also referred to as human parainfluenza virus 4, a respiratory pathogen from the *Paramyxoviridae* family, was the only negative-sense single-stranded RNA (ssRNA(-)) virus identified, while rotavirus (*Sedoreoviridae* family) was the only double-stranded RNA (dsRNA) virus detected. Other viral genome structures included single-stranded RNA Reverse Transcriptase (ssRNA-RT) (*Retroviridae*), double-stranded DNA (dsDNA) (*Adenoviridae* and *Herpesviridae*), and single-stranded DNA (ssDNA) (*Anelloviridae* and *Parvoviridae*) (Table S2).

Viruses that are commonly implicated in gastroenteritis in infants and young children were the most abundant. These included sapoviruses (SaVs), noroviruses (NoVs), enteroviruses (EVs), cardioviruses (CaVs), astroviruses (AstVs), adenoviruses (AdVs), parechoviruses (PeVs), rotaviruses (RVs), and cosaviruses (CoSVs). Slightly over one million of the eukaryotic viral reads were assigned to sapovirus and enterovirus C. These were followed by mamastrovirus 1, human mastadenovirus F, norovirus, parechovirus A.

##### 2.4.1.1 Changes in eukaryotic gut virome composition over time

**Fig. 4:**
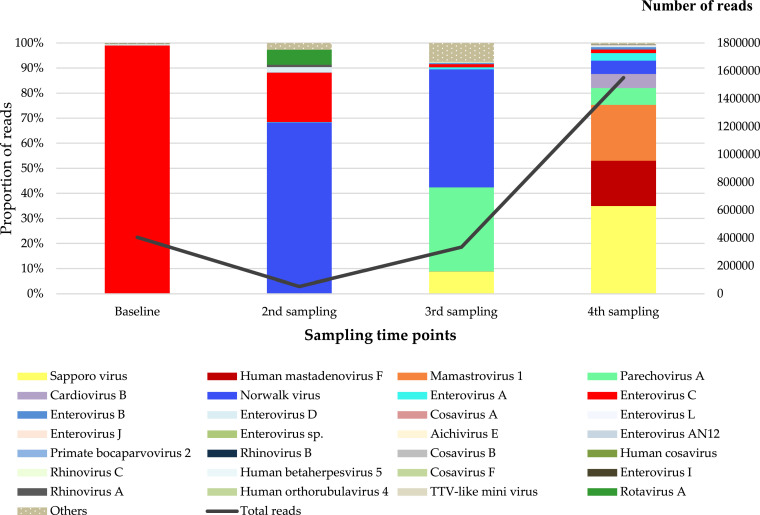
Compositional changes and dynamics of the infants’ gut virome over four time points. Baseline samples were collected during the first week of birth (0–7 days); 2nd sampling was done between 6 and 8 weeks of age; 3rd and 4th follow-up samples were collected at 16–20 weeks, and 24–26 weeks of age, respectively. The grey line represents the number of reads at each of the four time points.

The composition of viral communities at each of the four time points, and the changes over time were assessed. At baseline, a total of 404 255 viral reads were recovered, and 99 % of these reads were assigned to species enterovirus C, from the genus *Enterovirus*, family *Picornaviridae,* making it the most abundant virus detected during the first collection time point (Fig. 4). Further investigation of the detected enterovirus C genomes revealed a range of enterovirus C subtypes including human poliovirus vaccine strains. It is important to note that the infants were immunised with oral polio vaccine (OPV) shortly after birth, which therefore suggests the detection of vaccine-derived polio.

In the second time point, there was a nearly 8-fold decrease in total eukaryotic viral reads, as illustrated by the grey line in Fig. 4. We also observed a reduction in enterovirus C during this period, replaced by the predominance of norovirus, in the family *Caliciviridae*, accounting for 68 % of the reads. We further noticed the emergence of rotaviruses in the second sample collection time-point, and, like polioviruses, this coincides with the immunization schedule of the live-attenuated Rotarix vaccine, administered at 6 weeks of age.

Following the decrease in viral reads, there was an exponential increase in eukaryotic viral reads continuous from the second to the fourth time point. This upward trend was characterised by an expansion in virome composition with more viral diversity observed at 16–20 weeks and 24–26 weeks, respectively. While norovirus continued to dominate in the third time point, there was also elevated detection of parechovirus A, classified under the family *Picornaviridae*, contributing approximately 34 %, and the emergence of sapovirus (family *Caliciviridae*).

The infants’ eukaryotic virome became richer in the final collection time point, with a diverse population of viruses being detected. The viral reads increased from 334 378 to 1 550 512 from the third to fourth sampling time point (Fig. 4). Having emerged in the third time point, sapovirus virus became the most predominant viral species by age 24–26 weeks, accounting for a third of the viral reads. This was followed by mamastrovirus 1 species from the family *Astroviridae*, contributing 22 % of the eukaryotic virome in the fourth time point. We further detected a significant proportion of reads assigned to human mastadenovirus F, a member of the family *Adenoviridae*. Whilst norovirus virus and parechovirus reads were persistently abundant, other viruses detected in the fourth sample collection time point included various members of the *Picornaviridae* family such as enterovirus A, B, C, D, cardiovirus B, human cosaviruses, as well as respiratory viruses, human rhinovirus A, B, C (Fig. 4).

##### 2.4.1.2 Detection frequency of common human viruses and plant viruses

**Fig. 5:**
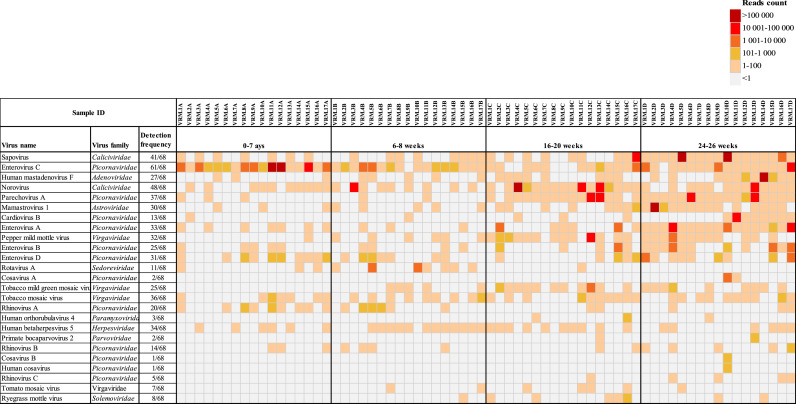
Heat map showing the reads distribution and detection frequency of important human pathogens and plant viruses across the 68 samples from 17 infants.

We investigated the prevalence of viruses of clinical significance, and that of plant viruses detected in stool samples of infants. Based on our analysis, enterovirus C was the most frequently detected pathogen, present in 61/68 (90 %) samples. This was followed by sapoviruses and noroviruses, with detection rates of 70 % and 60 %, respectively (Fig. 5). Other important human pathogens including masadenovirus F, mamastrovirus 1, enterovirus D, enterovirus A had prevalence rates between 40 % and 49 %, while human betaherpesvirus 5 and parechovirus A were detected in 50 % and 54 %, respectively. The rest of the viruses shown in Fig. 5 had relatively low detection rates, with cosaviruses, primate bocaparvovirus 2, and orthorubulavirus 4 being among the least detected (<5 %) (Fig. 5). While several viral agents began to emerge in later stages, many of the viruses were present in the stool samples collected within the first week and continued to be detected throughout. Looking into specific time points, enterovirus C was the only virus present in all 17 (100 %) infants at baseline, with norovirus being the second most prevalent, detected ten times (59 %) (Fig. 5).

In the second time point, enterovirus C continued to dominate, with human betaherpesvirus 5 showing a two-fold increase in prevalence by 6–8 week of age. The third time point samples were characterized by the dominance of norovirus and parechovirus A, the latter exhibiting a sharp increase in prevalence from 12 % in the second time point to 70 % in the third collection time point. Conversely, enterovirus C was still highly detected despite being cleared in some samples (Fig. 5). Remarkably, an increase in prevalence was observed for several viruses by 24–26 weeks of age. Sapovirus, parechovirus A, and enterovirus A all had 100 % detection rates in the fourth time point. Moreover, norovirus and mamastrovirus 1 were detected at frequencies of no less than 70 %, respectively. We also noted a continuous increase in the detection frequency of human mastadenovirus F over time, from about 18 % at baseline to over 60 % in samples collected between 24 and 26 weeks (Fig. 5). Of note, 99.8 % of all mastadenovirus F reads were detected in one sample at the fourth collection time-point.

In addition, plant viruses of the *Virgaviridae* family were well represented, with tobacco mosaic virus being the most prevalent, identified in more than 50 % of the samples overall. Pepper mild mottle virus followed at 47 %, exhibiting the highest detection frequency in the third and fourth sampling time points, while another diet-associated species, tomato mosaic virus, was only sporadically identified (Fig. 5).

##### 2.4.1.3 Co-occurrences of common gastroenteritis-associated viruses

**Fig. 6:**
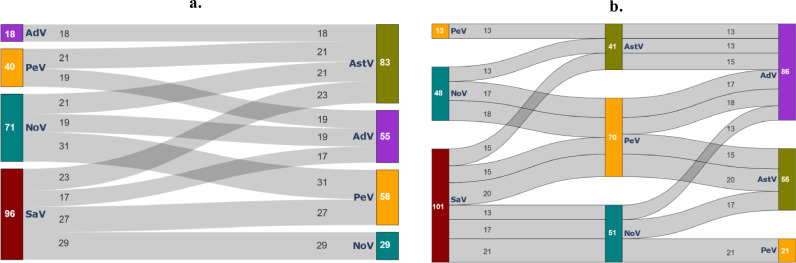
Sankey diagram depicting the dual (a) and triple (b) co-occurrences of gastroenteritis-associated viruses. NoV = norovirus; SaV = sapovirus; PeV = parechovirus; AstV = astrovirus; AdV = adenovirus.

As shown in Fig. 5, all 17 infants shed more than one type of enteric virus at each of the four sampling time points. The most common co-occurrence of gastroenteritis-associated viruses observed was with norovirus, parechovirus, and sapovirus (Fig. 6a and b). Dual co-occurrences of sapovirus-parechovirus, sapovirus-norovirus, and norovirus-parechovirus were observed in 27/68 samples (39.7 %), 29/68 samples (42.6 %) and 31/68 samples (45.6 %) of the samples, respectively. Furthermore, each of these viruses were also frequently observed in combination with astrovirus, as well as adenovirus.

#### 2.4.2 Bacteriophages

**Fig. 7:**
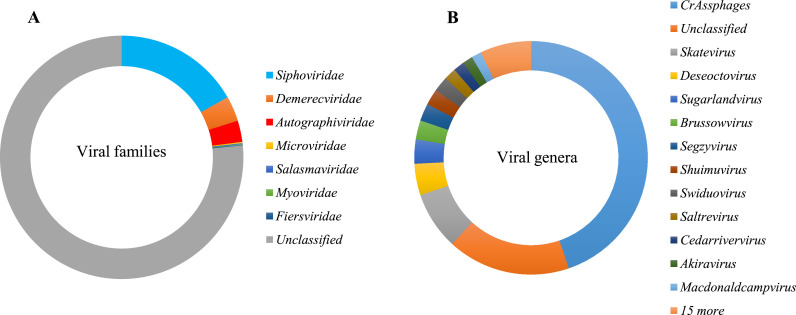
Taxonomic distribution of reads assigned to bacteriophages classified into different viral families (A) and viral genera (B).

In contrast to most gut virome metagenomics studies (Lim, Wang and Holtz, 2016; Pannaraj et al., 2018; Taboada et al., 2021), bacterial viruses were detected at relatively low abundances compared to eukaryotic viruses. Of the 60 143 reads assigned to bacteriophages, only 25 % could be classified into seven different viral families, while 75 % were unclassified at family taxonomic level (Fig. 7A and B). When investigating the temporal dynamics of the phage communities, very few viruses were present after birth, but rapidly emerged by six weeks of age, after which there was a continuous decline in abundance over time. In comparison to the eukaryotic viral populations, a low abundance and diversity was observed in earlier stools, increasing in richness and abundance with age.

Of the identified families, *Siphoviridae* was the most abundant (17 %), while *Demerecviridae* and *Autographiviridae* were equally represented at an abundance of 3 %, respectively. Other phages present at low abundances included family *Microviridae, Salasmaviridae, Myoviridae*, and *Fiersviridae* (Fig. 7). Except for *Microviridae* and *Fiersviridae*, the rest of the families belong to the order *Caudovirales*, which represents the largest group of temperate bacteriophages, dominating the gut bacteriophage communities.

At genus level, the infant gut phageome was dominated by crAssphages, unclassified members of the order *Caudovirales*, accounting for almost 45 % of the bacteriophage reads. The remaining reads were distributed among numerous genera of phage populations, comprising, among others, *Skatevirus* (8 %), *Deseoctovirus* (4 %), *Sugarlandvirus* (4 %), and *Brussovirus* (3 %), while for a considerable fraction of reads (17 %), the genus could not be assigned (Fig. 7).

Strikingly, 99.8 % of the reads identified as crAssphages were detected in faecal samples collected from one infant. These crAssphages were absent in the baseline sample, then rapidly emerged in the second sample (6–8 weeks), before decreasing in the third and fourth time points. In other infants, crAssphage sequences were sporadically detected in some samples, with none of the samples having more than five reads.

## 3 Discussion

Overall, the infant gut virome was dominated by eukaryotic viruses, consisting primarily of human enteric viruses and non-human dietary viruses. In contrast to common reports from some enteric virome studies (Breitbart et al., 2008; Reyes et al., 2010; Minot et al., 2012; Aggarwala, Liang and Bushman, 2017; Van Espen et al., 2021), the bacteriophage populations were present at relatively low abundance. These variations in virome composition may arise due to the differences in sample processing methods, including virome enrichment procedures, virus identification tools and bioinformatics approaches employed in different virome studies (Kleiner, Hooper and Duerkop, 2015; Shkoporov et al., 2019; Privitera et al., 2022), as there is no standardised procedure for viral metagenomic characterisation.

Similar to our previously published pilot study analysing the enteric RNA virome of a small cohort of South African infants (Mogotsi et al., 2020), the gut virome of infants in the current study was dynamic and diversified over time, with the eukaryotic viral species becoming more prominent with age. Despite the absence of clinical symptoms (i.e., diarrhoea, vomiting, or fever) in the infants under investigation during the sampling period, there was a high abundance of gastroenteritis-associated viruses in this study. Of the reads assigned to eukaryotic viruses, *Caliciviridae* and *Picornaviridae* families were the most predominant, an observation that is consistent with similar virome studies (Taboada et al., 2021; Walters et al., 2023), accounting for over 70 % of the eukaryotic viral reads.

Sapoviruses (SaVs) and noroviruses (NoVs), both members of the *Caliciviridae* family, were the most commonly occurring pathogens, detected from as early as less than one-week post-partum (Baker et al., 2021; Valcarce et al., 2021). Although SaV is commonly linked to outbreaks and sporadic occurrences of gastroenteritis in children (Hassan-Ríos et al., 2013; Chhabra et al., 2014; Iritani et al., 2014; Oka et al., 2015; Hergens et al., 2017; Wang et al., 2018; Valcarce et al., 2021), asymptomatic shedding of SaV has also been documented (Bucardo et al., 2012; Platts-Mills et al., 2018; Pitkänen et al., 2022), which can last for up to five weeks post infection (Oka et al., 2015; Sánchez et al., 2018). This indicates that the most common aetiologic agents of gastroenteritis in paediatric subjects may be found in asymptomatic carriers, serving as potential reservoirs for acute gastroenteritis (AGE), and thereby increasing the likelihood of viral transmission and outbreaks.

In South Africa, a hospital-based study investigating the prevalence of SaVs in children under five years with acute diarrhoea reported SaV detection in about 11 % of diarrhoea-related deaths, with two of the fatalities having SaV as a sole pathogen (Page et al., 2016). It was further reported that seven of the children who died had no flushing toilets, suggesting lack of proper sanitation as one of the risk factors for infection by enteropathogens. The study also highlighted the possible contribution of overcrowding in the transmission of SaV (Page et al., 2016), which may have also played a role in the current study as most infants lived with their parents, siblings/ and or other family members in either a rented backyard room, a shack or a family house with three or more rooms. Moreover, several studies from Africa have reported on the detection of SaVs in environmental samples such as wastewater and sewage (Kiulia et al., 2010; Murray, Mans and Taylor, 2013; Varela et al., 2018). Although the current study is based on infants who are not directly exposed to the environment, indirect contact through elder siblings or parents may have created an avenue for the faecal-oral transmission of SaVs.

In line with our observation, literature has reported on the prevalence of NoV in asymptomatic subjects (Kabue et al., 2016; Qi et al., 2018). Interestingly, a recent study investigating the prevalence of SaV, NoV and rotavirus (RV) in the faeces of Finnish children reported asymptomatic NoV infections to be higher in children <6 months old (Pitkänen, Markkula and Hemming-Harlo, 2022), similar to our participants’ age group. Locally, results from a multi-country longitudinal study examining NoV epidemiology, showed a prevalence of asymptomatic carriage to be 30 % in South African infants (Rouhani et al., 2016), further supporting our findings. Although the presence of asymptomatic NoV infection has been speculated to result from long-term shedding from a previous symptomatic episode (Qi et al., 2018), none of our study participants had diarrhoea. Therefore, a true asymptomatic infection is probable, potentially facilitated by environmental contamination, nosocomial transmission and/ or transmission from family members.

Within the *Picornaviride* family, a wide diversity of viruses was detected, with the predominance of species from the genus *Enterovirus* and *Parechovirus*. The high detection rate of enterovirus C, especially in baseline samples prompted further analysis, which revealed the presence of vaccine-associated poliovirus. This could be attributed to the vaccination of infants with oral poliovirus vaccine (OPV), administered at birth in South Africa.

Despite being key drivers of polio eradication, the live-attenuated strains in the OPV vaccines can replicate in the human gut, giving rise to mutations in the vaccine strains (Burns et al., 2014). Such an event can revert into a transmissible and virulent strain capable of causing paralytic poliomyelitis if allowed to circulate. The emergence of circulating vaccine-derived poliovirus (cVDPV) has been reported in Africa (Uwishema et al., 2022). Other members of the genus *Enterovirus* detected in high abundance included enterovirus (EV) A, B, D, K, L, AN12, unassigned enterovirus species, and respiratory viruses including rhinovirus A, B, and C.

Parechovirus (PeV) A, which belongs to the *Picornaviridae* family, was sporadically detected in the first and second sampling time points and in high frequencies during the last two sample collection time points, a pattern similar to that of SaV and NoV. Although PeV infections have been associated with life-threatening disorders including gastroenteritis, aseptic meningitis, acute flaccid paralysis (AFP), and encephalitis, their detection in healthy infants support the evidence that PeV infections are commonly subclinical (Ito et al., 2004; de Crom et al., 2016; Zhang et al., 2022).

Despite their relatively low detection rate (∼19 %), cardiovirus (CaV) B viral reads were significantly high and aggregated mostly in the last collection time points. These members of the *Picornaviridae* family were previously believed to primarily infect rodents (Pritchard, Strom and Lipton, 1992). In 2007, a novel human cardiovirus, known as Saffold virus (SAFV), was isolated from stool specimen of a child experiencing fever (Jones et al., 2007). SAFV was later detected in children with non-polio acute flaccid paralysis, as well as infants with respiratory or gastrointestinal illnesses (Abed and Boivin, 2008; Drexler et al., 2008; Blinkova et al., 2009). Despite their presence in paediatric patients with gastroenteritis (Li et al., 2017), as well as in environmental samples, there are not routine screening of SAFV in clinical samples (Fernandez-Cassi et al., 2018; Ferraro et al., 2019).

Cosaviruses (CoSV), members of the *Picornaviridae* family, were originally discovered in stool samples of individuals suffering from acute flaccid paralysis (AFP) and their healthy contacts (Kapoor et al., 2008). Since then, it has been reported in children with and without diarrhoea in several countries across the world (Holtz et al., 2008; Dai et al., 2010; Stöcker et al., 2012; Khamrin and Maneekarn, 2014). In Tunisia and India, CoSV was reported in cases of non-polio AFP at a prevalence of above 30 %, respectively (Maan et al., 2013; Rezig et al., 2015). Another study in Bangladesh reported human CoSVs to be the most prevalent enteric virus (∼40 %) in asymptomatic infants’ stools, (Okitsu et al., 2020). In the present study, CoSV A, B and F were detected in two infants. A related longitudinal gut virome study in Ghana reported the detection of novel strains of CoSV A in the stool of asymptomatic infants, with the potential role in limiting immunogenicity of rotavirus vaccines (Kim et al., 2022).

Astroviruses (AstVs), from the family *Astroviridae*, are classified into two genera based on their hosts of origin namely, *Mamastrovirus* and *Avastrovirus*, the former infecting mammals and the latter infecting avian species (Bosch, Pintó and Guix, 2014). In our study, *Astroviridae* (Mamastrovirus 1) was the third most abundant viral family in the infants’ eukaryotic gut virome. Human astroviruses have long been considered important causative agents of acute gastroenteritis (AGE), after rotavirus (RV) and NoV, predominantly affecting the paediatric population (Bosch, Pintó and Guix, 2014). Although AstV-induced diarrhoea is less severe than that caused by RV or NoVs, it can sometimes result in hospitalisation. In addition, asymptomatic infections have also been reported in a some African countries including Kenya and Gambia (Meyer et al., 2015), and South Africa (Khumela et al., 2021). Moreover, Nadan and colleagues reported significant association between source of water and AstV infection in South African children, with higher detection frequencies in patients using outdoor water sources than indoor taps (Nadan et al., 2019), showing the important role of socio-economic status in the transmission of enteric pathogens.

Rotavirus (RV) (family *Sedoreoviridae*) is the predominant causative agent of diarrhoea-associated morbidity and mortality in children under five years of age, and the burden of rotavirus disease is high in sub-Saharan Africa and South Asia (Troeger et al., 2018). Unlike other gastroenteritis-causing viruses reported in this study, RV was present at low abundance, and it was mainly detected at 6–8 weeks of age, coinciding with the first dose of oral rotavirus vaccination schedule. Analysis of the capsid viral proteins (VP4 and VP7) revealed mostly G1P[8] genotype combination, suggesting potential shedding of the Rotarix G1P[8]-containing vaccine strain. The shedding of rotavirus vaccine in human stool has long been documented (Ruiz-Palacios et al., 2007), while in 2014, Hsieh and colleagues demonstrated that rotavirus vaccine was shed in more than 80 % of vaccine recipients few days after administration of the first dose (Hsieh et al., 2014). Comparable observations were made in South African infants, where vaccine shedding was higher in the first dose and was significantly reduced in the second dose of monovalent vaccine (Magwira et al., 2020). More recently, a cohort-based longitudinal study from a low-resource setting indicated an increase in the prevalence of G1P[8] strains from 3 % prior to the first dose of rotavirus vaccine to 50 % after administration, and 25 % after the second dose (da Cunha et al., 2023). This is consistent with our results, which reported almost no detection of RVs after the second dose. In addition, the short duration of shedding of vaccine-derived strains, estimated to last for eight days, could also be a factor (da Cunha et al., 2023).

Human adenoviruses (HAdVs), classified under the family *Adenoviridae* are a group of dsDNA viruses causing a range of morbidities in humans, including respiratory illnesses, gastroenteritis, pneumonia, and conjunctivitis. (Lynch and Kajon, 2016). Currently, 51 serotypes of human AdVs have been identified, with more than 100 genotypes described, and classified into seven species (A-G) (Kajon, Weinberg and Spindler, 2019; Lion, 2019). Among these, species F, which comprises types 40 and 41, have long been recognised as aetiologic agents of gastroenteritis in children (Kotloff et al., 1989).

In the current study, human mastadenovirus F was the only human AdV species, detected in a total of 27 samples from 14 infants, across the four time points. While sporadic detections of human mastadenovirus F was observed at baseline, second, and third time points, over 99 % of the reads were recovered from a single specimen collected at 24–26 weeks. This sample belonged to an infant with no clinical symptoms, delivered vaginally, was exclusively breast-fed, and with no record of human immunodeficiency virus (HIV) exposure. Although there is existing evidence of asymptomatic carriage of human AdV, the overwhelming abundance of AdV reads from a single infant, in the absence of clinical manifestations is concerning. This could, however, suggest a potentially newly established infection in this infant, and thus warrant further investigation.

A birth cohort study with over 2000 children under two year olds, from eight low- to middle-income countries including South Africa, reported AdV 40/41 as the second highest detected pathogen in diarrhoeal cases, following rotavirus (Platts-Mills et al., 2015). Another study examining the co-infection of enteric pathogens in South African children with diarrhoea reported human AdV 40/41 as the most frequently detected viral pathogen (Potgieter et al., 2023). As demonstrated in this study, the prevalence of AdV 40/41 in infants stool samples was quite significant. Considering its role in diarrhoeal disease burden, AdV 40/41 seems to be an attractive target for vaccine development, since none is currently available.

Apart from AdVs, other DNA viruses detected in this study included members of the *Anelloviridae* family, i.e., torque teno virus-like mini virus (TTMV) in the genus *Betatorquevirus*. Despite having not been associated with human diseases, anelloviruses are a large group of viruses frequently detected in healthy children (Reyes et al., 2015; Lim, Wang and Holtz, 2016; Maqsood et al., 2019; Liang, Conrad, et al., 2020). Thus, their detection in infants’ gut is not unexpected, as this could imply anelloviruses are key components of the human virome. A recent study analysing the gut virome of infants in their first year of life reported various TMMV species in up to 80 % of their specimen (Taboada et al., 2021). In the current study, about 98 % of anellovirus sequences were found in one infant in a sample collected between 16 and 20 weeks. A study by Lim and colleagues reported an expansion in anellovirus richness in infants gut during the second half of the first year of life (Lim et al., 2015), suggesting that increasing environmental exposure could be a role player. Although anelloviruses have previously been associated with immunosuppression (Vlaminck et al., 2013), our sequences were largely recovered from an infant with no exposure to HIV, thus, the abundance of anelloviruses in this infant warrants further investigation.

Primate bocaparvovirus 2 and human betaherpesvirus 5 in the families *Parvoviridae* and *Herpesviridae*, respectively, were other DNA eukaryotic viruses detected in these infants. *Bocaparvovirus*, a genus of the subfamily *Parvoviridae*, have been identified in humans and non-human primates. Primate bocaparvovirus 2 include human bocavirus (HBoV), which was first identified in 2005 in respiratory secretions of children presenting with respiratory symptoms (Allander et al., 2005), and subsequently detected in human faecal specimens. In asymptomatic subjects, HBoV is usually present in combination with other viruses, with type 2 being the most prevalent in stool samples of children (Arthur et al., 2009; Kapoor et al., 2009, 2010). Co-infection of HBoV with other respiratory and gastrointestinal viruses, such as rhinovirus, norovirus, adenovirus and rotavirus has been reported (Arthur et al., 2009; Kapoor et al., 2009). In the current study, bocavirus sequences were detected in only one infant, persisting from 16 to 20 weeks throughout to 24–26 weeks of age, and like previous studies, co-infection with rhinovirus C, norovirus, adenovirus and sapovirus, among others was also observed. The clinical relevance of HBoV in this study is unknown, and association with gastroenteritis has also not been elucidated by previous studies.

Human betaherpesvirus 5, also known as human cytomegalovirus (HCMV), is a ubiquitous member of the *Herpesviridae* family, reported to have a seroprevalence of approximately 40 % to 100 % in adults, characterised by age, socio-economic background, and geographic region (Cannon, Schmid and Hyde, 2010). HCMV is responsible for mild to asymptomatic infections in immunocompetent persons, however the virus is rarely cleared, resulting in latent infection (Reeves and Sinclair, 2008; Mocarski et al., 2013). Conversely, infection of immunocompromised persons, such as HIV positive individuals, can result in potentially life-threatening diseases and even death (Boeckh and Geballe, 2011).

In our study, human betaherpesvirus 5 or human cytomegalovirus was detected in all 17 infants at different time points, from as early as one week of age. Although the source of HCMV infection in this cohort is not clear, congenital transmission of HCMV during pregnancy, to the foetus, is well documented (Cannon, Schmid and Hyde, 2010; Mocarski et al., 2013; Marsico and Kimberlin, 2017), which can lead to mental retardation, and is responsible for 25 % of all cases of hearing loss in children (Grosse, Ross and Dollard, 2008). In African settings, the rate of congenital HCMV infection seems to be higher, with strong association to maternal HIV infection (Mwaanza et al., 2013). This is further supported by findings from a South African study, which demonstrated a high rate of congenital HCMV infection in HIV-exposed neonates, also linked to maternal immunosuppression (Manicklal et al., 2014). In our study, we did not identify any correlation between HCMV and HIV.

Hygiene practices like frequent washing of hands have been recommended as precautionary measures for the prevention HCMV infection, however, there is little awareness in the public about HCMV (Cannon and Davis, 2005). Therefore, efforts must be intensified to educate the public about the importance of HCMV, particularly women of child-bearing age. In addition, we propose routine prenatal screening of such viruses, which are potentially harmful to the foetus and neonates, should be enforced.

Plant-infecting viruses are prevalent in human populations, including in the gut of infants (Zhang et al., 2006; Lim et al., 2015; Aguado-García et al., 2020; Mogotsi et al., 2020; Rivera-Gutiérrez et al., 2023). Our results revealed a high abundance of sequencing reads homologous to plant viruses, predominantly from the family *Virgaviridae* (pepper mild mottle virus (PMMV), tobacco mild green mosaic virus (TMGMV), tobacco mosaic virus (TMV), and tomato mosaic virus (ToMV), and one species from family *Solemoviridae* (ryegrass mottle virus (RGMoV)). Our study confirms findings from Aguado-García and colleagues, who described the frequent presence of plant viruses, dominated by the family *Virgaviridae*, in the gut of exclusively breast-feeding infants, two weeks after birth (Aguado-García et al., 2020). The presence of diet-associated plant viruses such as PMMV and ToMV in infants who are not exposed to solid food could suggest vertical transmission from mother to infant as a potential source of these viruses, or possibly, contact between infant and siblings or other family members.

Several non-human mammalian viruses were detected, including hunnivirus A, aichivirus E, torchivirus A, mischivirus D, and crohivirus A, all of which belong to the family *Picornaviridae*. Hunnivirus was first detected in 1965 from sheep cell cultures in Northern Ireland, and more recently, hunnivirus has been detected in cattle, sheep, and rats (Reuter et al., 2012; Firth et al., 2014; Zhang et al., 2021). A recent study discovered a novel hunnivirus in a cat with diarrhoea in China (Lu et al., 2019), which raises an interesting question regarding its potential to be transmitted by pets. More intriguing, in another Chinese study, phylogenetic analysis revealed that rat hunnivirus sequence shared a common root with cat hunnivirus sequence, indicating possible inter-species transmission of hunnivirus between rodents and other animals. Since human are in constant contact with these animals, this poses a threat to public health (Zhang et al., 2021).

Torchivirus was previously isolated from terrestrial tortoises (Farkas et al., 2015), thus, the source of this virus in infants is unclear, and its clinical relevance in humans remains unknown. Only 33 sequences of torchivirus species D were detected in this study.

Aichivirus, a member of the genus *Kobuvirus*, was first detected in a faecal sample associated with the consumption of oysters during a gastroenteritis outbreak in Japan (Yamashita et al., 1991) and consists of six recently renamed species: Aichivirus A to F (Adams et al., 2017). Subtypes of Aichivirus A and Aichivirus B have been recognised as human pathonges responsible for outbreaks of gastroenteritis (Rivadulla, 2020). In this study, however, Aichivirus E, known to infect rabbits, was detected in the stool of infants.

Crohivirus was first detected in faecal specimen of wild shrews in Zambia by metagenomic analysis (Sasaki et al., 2015), and later in Cameroonian fruit bats (Yinda et al., 2017). The presence of crohiviruses in humans has not been reported before, however, potential spread from bats, which are commonly found near human residences and human gathering spaces cannot be ruled out.

Similarly, *Mischivirus* is another genus associated with bats (mischivirus A, B, and C) and foxhound (mischivirus D) (Lukashev et al., 2017; Norby et al., 2017). The detection of bat-infecting viruses in humans is concerning, as bats are considered natural reservoirs for a wide spectrum of viruses capable of zoonotic transmissions, including emergent human pathogens, such as SARS-CoVs, Ebola viruses and Marburg virus.

Existing evidence, from earlier to recent studies, supports the observation that the gut virome of infants is dominated by bacteriophages, potentially derived from the colonising gut bacteria, soon after birth (Breitbart et al., 2008; Beller et al., 2022). In this cohort of infants, however, the proportion of phage communities was almost 40-fold smaller than the eukaryotic virome. Our study reported nearly no detection of bacteriophages at birth, followed by a sharp increase in abundance few weeks later. These phage populations began to show a decline in abundance, and this contraction in richness was replaced by an expansion in the diversity of eukaryotic viral populations, reaching its highest density by the fourth time point. This expanding diversity of eukaryotic virome, over time, support the hypothesis that eukaryotic viruses are largely acquired through environmental exposure (Beller et al., 2022).

Although our findings confirm previous studies describing the predominance of crAssphages in the gut (Tamburini, Sherlock and Bhatt, 2018; Shkoporov et al., 2019), its high abundance in only one infant is questionable. First discovered in 2014 using the cross-assembly (crAss) approach, crAssphages are the most abundant and ubiquitous bacteriophages in the human gut (Dutilh et al., 2014). According to Tamburini and colleagues, crAssphages can be acquired in various ways, including vertical transmission from mother to infant, through faecal microbiota transplantation, as well as in immunocompromised hosts in hospital settings (Tamburini, Sherlock and Bhatt, 2018). Despite these multiple forms of acquisition and transmission, establishing the source of crAssphages in our study subject is more complex, and this is exacerbated by lack of disease association with crAssphages. Nonetheless, some authors believe that crAssphages prey on bacteria of the phylum Bacteroidetes (Dutilh et al., 2014; Yutin et al., 2018), which are commonly present in infants within the first weeks of birth (Hill et al., 2017).

Certainly, gut microbial colonisation in infants is a crucial, yet intricate, process, characterised by minimal diversity at birth, increasing with time. There are several factors known to influence the gut virome composition. In our study, we investigated the impact of birth mode, diet, and gestational age to evaluate any differences in the infants’ viromes. We did not notice any discernible variation in the gut virome diversity or composition in infants. Our study was, however, not suited to effectively assess the impact of birth since there were only three infants born via C-section compared to 14 born by vaginal delivery. With regards to diet and gestation age, all study infants were exposed to breastmilk and were not yet introduced to solid feeding during the study period. This lack of differences in the infant's diet was a limiting factor in examining the influence of diet on the gut virome composition. Similarly, only two of the infants were pre-term (delivered before 37 weeks), and, as such, their viromes were not distinguishable to those delivered after 37 weeks.

A study of this magnitude is rarely without limitations. Cohort-based longitudinal virome studies conducted over a longer period (e.g., at least two years) are required to precisely assess the compositional changes even after introduction of solid food, and the impact of other factors like pre-school settings. Apart from the missing demographic information, such as HIV exposure for some of the participants, the ratio between the different birth modes was not adequately balanced to enable fair comparison of virome composition. Several participants were lost to follow-up due to restricted movement during Covid-19 lockdowns, consequently reducing the sample size.

## 4 Conclusions

This study provides an overview, and baseline knowledge on how the gut virome of mainly asymptomatic infants is assembled over time, which is a significant step towards understanding the dynamics and biogeography of viral communities in the infant gut. Data emanating from this study proves, beyond doubts, that even in the absence of clinical symptoms, the infant gut is highly colonised by a spectrum of medically important and potentially harmful viruses from as early as few days after birth. These results further reveal the complex nature of microbial co-infections in infants, persisting for several weeks and months. These observations highlight the importance of enteropathogen screening at an early age, to close the existing gaps and improve the effectiveness of currently available treatment. Moreover, the detection of heterogeneous pathogens of unknown origin in stools of infants underscores the need to extend sampling to mothers to better understand transmission patterns of some viruses. It is crucial that future virome studies are designed to precisely elucidate the role of vertical transmission of microbes present in the gastrointestinal tract of infants. Additionally, it would be of interest for future studies to explore factors that could potentially be contributing to the persistence of certain viruses, from birth, in the gut of healthy infants, and prolonged shedding in faeces, as these could result in severe infections.

As most enteric pathogens are transmitted via the faecal-oral route, implementing household-level interventions to improve the quality of water, sanitation, and hygiene would be highly beneficial. However, to drastically minimize the burden of diarrhoeal diseases especially in low- to middle-income countries, efforts to develop vaccines for viral agents like adenovirus 40/41 must be intensified.

In conclusion, this study has demonstrated the extent and persistence of viral infections occurring in early childhood, and while most of the detected enteric viruses possess the potential to cause severe illnesses, they mostly appear to induce no symptoms in this paediatric cohort. Nonetheless, since all infants were breastfeeding during their first six months, the maternal antibodies from breast milk may have offered protection against severe symptoms.

## 5 Materials and methods

### 5.1 Ethics statement

The study was reviewed and approved by the Free State Department of Health and the University of the Free State Health Sciences Research Ethics Committee (HSREC), under ethics number **UFS-HSD2020/0327/2710**.

### 5.2 Study population and specimen collection

This cohort-based longitudinal study was conducted at three public hospitals, namely, Pelonomi Regional Hospital, National District Hospital, and Mangaung University Community Partnership Programme (MUCPP) Health Center, between October 2020 and August 2022. All the study sites are based in the capital city of Bloemfontein within the Mangaung Metropolitan Municipality, Free State Province, located in central South Africa. Expectant women and mothers who had given birth within the past seven days were recruited from the maternal wards of the above-mentioned hospitals. Written informed consent was obtained from all interested participants after detailed information about the research study was provided to them. Following enrolment, clinical and demographic metadata was collected, in a form of a questionnaire, including, but not limited to, age, gender, birth weight, gestational age, birth mode, and HIV exposure (see Supplementary Table S1 for full demographic data). Faecal samples were longitudinally collected from neonates at four-time intervals, with the first sample collected in less than seven days post-partum. Follow-up sample and data collection were carried out at 6–8 weeks, 16–20 weeks, and 24–28 weeks. The collected faecal samples were transported, in ice-boxes, to the University of the Free State-Next Generation Sequencing (UFS-NGS) Unit, Bloemfontein, South Africa and stored at −80 °C until further processing.

### 5.3 Virus enrichment and nucleic acid extraction

Collected faecal specimens were processed using the **N**ovel **E**nrichment **T**echnique **O**f **V**iromes (NetoVIR) protocol (Conceição-Neto et al., 2015), with modifications. Study subjects were assigned identification names VRMx (*x* = participant number), with alphabets A, B, C or D used to represent each of the four sample collection time points i.e., baseline, second, third, and fourth collection, respectively. Briefly, faecal material (∼100 mg) was resuspended in 1 mL of phosphate-buffered saline (PBS) (Sigma-Aldrich, P4417–50TAB) and homogenised at 3 000 rpm for 1 min using a Beadbug microtube homogeniser (Benchmark Scientific, Sayreville, NJ, USA) to prepare a 10 % faecal suspension. This was followed by centrifugation at 13 500 rpm for 3 min using Prism microcentrifuge (Labnet, Edison, NJ, USA) to pellet cellular debris. At least 300 µL of supernatant was collected and filtered using a sterile 0.45 µm syringe filter (GVS, Bologna, Italy) to eliminate larger-sized eukaryotic and bacterial cells. To digest the extracellular free-floating nucleic acids, 130 µL of the filtrate was treated with a combination of 2 µl benzonase nuclease (Merck Millipore, 70,746–3) and 1 µL micrococcal nuclease (New England Biolabs, M0247S) in 7 μL buffer consisting of 1 M Tris, 100 mM CaCl_2_ and 30 mM MgCl_2_, pH 8) for 2 h at 37 °C. The reaction was stopped by adding 7 μL of 0.5 M EDTA. Nucleic acid extraction (RNA and DNA) was done using QIAamp Viral RNA Mini kit (Qiagen, 52906), without carrier RNA.

### 5.4 Random amplification

Whole transcriptome amplification was performed using QIAseq FX Single Cell RNA Library Preparation Kit (Qiagen, 180,735). The purity of amplified DNA was determined on µlite BioDrop spectrophotometer (BioDrop, Cambridge, UK), while DNA concentrations were measured on Qubit 3.0 fluorometer using Qubit dsDNA High Sensitivity Assay kit (Thermo Fischer Scientific, Q32854).

### 5.5 DNA library preparation and next generation sequencing

Genomic libraries were prepared with the QIAseq FX Single Cell RNA Library Preparation Kit (Qiagen, 180,735), uniquely indexed, and purified using Ampure XP beads (Beckman Coulter, A63881). Library fragment sizes were determined on Agilent 2100 Bioanalyzer (Agilent Technologies, Santa Clara, CA, USA), using dsDNA High Sensitivity Assay kit (Agilent, 5067–4626). Indexed libraries were fluorometrically quantified on Qubit, normalised to equimolar concentrations of 4 nM, and pooled into a 1 mL microcentrifuge tube. The library pool was denatured with a freshly prepared 0.2 N sodium hydroxide (NaOH), followed by dilution with hybridisation buffer (HT1) to a final concentration of 8pM. Prior to NGS, the library was spiked with 5 % PhiX Control V3, and sequenced on the Illumina MiSeq platform (Illumina, San Diego, CA, USA) for 300 cycles, using a MiSeq reagent kit V3 (Illumina, 15,043,894) to generate 2 × 150 bp paired-end reads.

### 5.6 Viral metagenomic analysis

Raw demultiplexed reads obtained from the MiSeq Illumina platform were assessed for quality FastQC (Andrews, 2010). Downstream analysis of the metagenomic data entailed taxonomic assignments and classification of the sequencing reads using Kraken V2 (Wood, Lu and Langmead, 2019). Computation of species relative abundances was done using Bayesian *Re*-estimation of Abundance with Kraken (Bracken) (Lu et al., 2017) and visualisation of microbial abundance profiles using Pavian R package v 0.8.4 (Breitwieser and Salzberg, 2020). Additional analysis including calculation of averages and plots were done on Microsoft Excel version 2016.

## CRediT authorship contribution statement

**Milton Tshidiso Mogotsi:** Writing – original draft, Visualization, Validation, Project administration, Methodology, Investigation, Formal analysis, Data curation. **Ayodeji Emmanuel Ogunbayo:** Writing – review & editing, Methodology, Investigation. **Phillip Armand Bester:** Writing – review & editing, Software, Methodology, Formal analysis. **Hester Gertruida O'Neill:** Writing – review & editing, Supervision, Project administration. **Martin Munene Nyaga:** Writing – review & editing, Supervision, Resources, Project administration, Funding acquisition, Conceptualization.

## Declaration of competing interest

The authors declare that they have no known competing financial interests or personal relationships that could have appeared to influence the work reported in this paper.

## Data Availability

Data will be made available on request.

## Appendix Supplementary materials

Image, application 1
