# Supplementary material for: Longitudinal analysis of the enteric virome in paediatric subjects from the Free State Province, South Africa, reveals early gut colonisation and temporal dynamics
Source: Virus Res. 2024 Jun 1;346:199403. doi: 10.1016/j.virusres.2024.199403 (PMC11169482; doi:10.1016/j.virusres.2024.199403)
Supplement: Supplementary file 1 [file mmc1.docx]

**Table S1**: Eukaryotic viruses detected from 68 infant faecal samples and the number of reads assigned to each virus.

| Family | Genera | Species | Genome structure | Host type | Number of reads |  |
| --- | --- | --- | --- | --- | --- | --- |
| *Picornaviridae* | *Enterovirus* | Enterovirus C | ssRNA(+) | Vertebrates | 436 498 |  |
|  |  | Enterovirus A | ssRNA(+) | Vertebrates | 50 016 |  |
|  |  | Enterovirus B | ssRNA(+) | Vertebrates | 16 770 |  |
|  |  | Enterovirus D | ssRNA(+) | Vertebrates | 12 794 |  |
|  |  | Enterovirus L | ssRNA(+) | Vertebrates | 1 855 |  |
|  |  | Enterovirus J | ssRNA(+) | Vertebrates | 787 |  |
|  |  | Enterovirus sp. | ssRNA(+) | Vertebrates | 477 |  |
|  |  | Enterovirus AN12 | ssRNA(+) | Vertebrates | 345 |  |
|  |  | Rhinovirus A | ssRNA(+) | Vertebrates | 1 096 |  |
|  |  | Rhinovirus B | ssRNA(+) | Vertebrates | 197 |  |
|  |  | Rhinovirus C | ssRNA(+) | Vertebrates | 103 |  |
|  |  | Enterovirus I | ssRNA(+) | Vertebrates | 63 |  |
|  | *Cardiovirus* | Cardiovirus B | ssRNA(+) | Vertebrates | 87 056 |  |
|  | *Cosavirus* | Cosavirus A | ssRNA(+) | Vertebrates | 2 057 |  |
|  |  | Cosavirus B | ssRNA(+) | Vertebrates | 113 |  |
|  |  | Human cosavirus | ssRNA(+) | Vertebrates | 111 |  |
|  |  | Cosavirus F | ssRNA(+) | Vertebrates | 56 |  |
|  | *Crohivirus* | Crohivirus A | ssRNA(+) | Vertebrates | 46 |  |
|  | *Dicipivirus* | Cadicivirus A | ssRNA(+) | Vertebrates | 1 098 |  |
|  | *Kobuvirus* | Aichivirus E | ssRNA(+) | Vertebrates | 417 |  |
|  | *Parechovirus* | Parechovirus A | ssRNA(+) | Vertebrates | 215 745 |  |
|  | *Hunnivirus* | Hunnivirus A | ssRNA(+) | Vertebrates | 5 793 |  |
|  | *Mischivirus* | Mischivirus D | ssRNA(+) | Vertebrates | 67 |  |
|  | *Torchivirus* | Torchivirus A | ssRNA(+) | Vertebrates | 33 |  |
| *Caliciviridae* | *Sapovirus* | Sapovirus | ssRNA(+) | Vertebrates | 570 976 |  |
|  | *Norovirus* | Norovirus | ssRNA(+) | Vertebrates | 275 395 |  |
| *Retroviridae* | *Gammaretrovirus* | Murine leukemia virus | ssRNA-RT | Vertebrates | 6 212 |  |
|  |  | Murine leukemia-related retroviruses | ssRNA-RT | Vertebrates | 202 |  |
|  | *Lentivirus* | Equine infectious anemia virus | ssRNA-RT | Vertebrates | 104 |  |
| *Adenoviridae* | *Mastadenovirus* | Human mastadenovirus F | dsDNA | Vertebrates | 280 219 |  |
|  |  | Platyrrhini mastadenovirus A | dsDNA | Vertebrates | 59 |  |
|  |  | Simian mastadenovirus C | dsDNA | Vertebrates | 57 |  |
| *Anelloviridae* | *Betatorquevirus* | TTV-like mini virus | ssDNA(-) | Vertebrates | 87 |  |
| *Astroviridae* | *Mamastrovirus* | Mamastrovirus 1 | ssRNA(+) | Vertebrates | 211 156 |  |
|  |  | Human astrovirus | ssRNA(+) | Vertebrates | 135 349 |  |
| *Paramyxoviridae* | *Orthorubulavirus* | Human orthorubulavirus 4 | ssRNA(-) | Vertebrates | 785 |  |
| *Parvoviridae* | *Bocaparvovirus* | Primate bocaparvovirus 2 | ssDNA | Vertebrates | 218 |  |
| *Dicistroviridae* | *Unclassified* | Big Sioux River virus | ssRNA(+) | Invertebrates | 122 |  |
| *Herpesviridae* | *Cytomegalovirus* | Human betaherpesvirus 5 | dsDNA | Vertebrates | 238 |  |
| *Sedoreoviridae* | *Rotavirus* | Rotavirus A | dsRNA | Vertebrates | 3 153 |  |
| *Virgaviridae* | *Tobamovirus* | Pepper mild mottle virus | ssRNA(+) | Plants | 19 785 |  |
|  |  | Tobacco mild green mosaic virus | ssRNA(+) | Plants | 1 798 |  |
|  |  | Tobacco mosaic virus | ssRNA(+) | Plants | 1 473 |  |
|  |  | Tomato mosaic virus | ssRNA(+) | Plants | 75 |  |
| *Solemoviridae* | *Sobemovirus* | Ryegrass mottle virus | ssRNA(+) | Plants | 348 |  |
| Total |  |  |  |  | 2 341 404 |  |

**Table S2**: Full demographic data of infants participating in the study.

| **No.** | **Participant ID** | **Area** | **Area type** | **Child's D.O.B** | **Sex** | **Hospital** | **Delivery mode** | **Gestational period (weeks)** | **Birth weight** | **HIV exposure** | **Diarrhoeal illness during sample collection** | **Feeding type** | **Started solid foods** | **Antibiotics exposure** | **1st collection (0-7 days)** | **2nd collection (6-8 weeks)** | **3rd collection (16-20 weeks)** | **4th collection (24-26 weeks)** | **House type** | **Running water** | **Flushing toilet** | **Electricity supply** | **Baby lives with** | **Source of income (salary/social grant/both)** |
| --- | --- | --- | --- | --- | --- | --- | --- | --- | --- | --- | --- | --- | --- | --- | --- | --- | --- | --- | --- | --- | --- | --- | --- | --- |
| 1 | VRM1 | Mangaung | Suburb | 2021/05/26 | F | Pelonomi | C-section | 39 | 3790 g | No | No | Exclusive breastfeeding | 6 months | Not specified | 2 | 8,4 | 18,6 | 25,9 | House | Yes | Yes | Yes | Parents and siblings | Salary |
| 2 | VRM2 | Mangaung | Township | 2021/06/03 | M | Pelonomi | Vaginal | 38 | 3140 g | No | No | Exclusive breastfeeding |  |  | 1 | 8,1 | 18,9 | 26,3 | Rented room | Yes | Yes | Yes | Parents and siblings | Both |
| 3 | VRM3 | Mangaung | Informal settlement | 2021/06/04 | F | MUCPP | Vaginal | 40 | 2180 g | No | No | Exclusive breastfeeding |  |  | 0 | 8,0 | 18,7 | 26,1 | Shack | Yes | Yes | Yes | Parents | Both |
| 4 | VRM4 | Mangaung | Formal settlement | 2021/06/24 | M | Pelonomi | Vaginal | 38 | 3210 g | No | No | Partial breastfeeding |  |  | 5 | 7,7 | 19,1 | 24,3 | House | Yes | Yes | Yes | Parents | Both |
| 5 | VRM5 | Mangaung | Township | 2021/06/24 | F | Pelonomi | C-section | 39 | Unknown | Yes | No | Exclusive breastfeeding |  |  | 6 | 7,7 | 19,7 | 24,3 | House | Yes | Yes | Yes | Parents and siblings | Social grant |
| 6 | VRM6 | Mangaung | Township | 2021/07/02 | F | Pelonomi | C-section | 34 | 2300 g | No | No | Partial breastfeeding |  |  | 3 | 6,0 | 18,6 | 24,1 | House | Yes | Yes | Yes | Parents and siblings | Social grant |
| 7 | VRM7 | Mangaung | Township | 2021/07/02 | F | MUCPP | Vaginal | 41 | 3100 g | Yes | No | Exclusive breastfeeding |  |  | 0 | 6,0 | 18,4 | 24,1 | Rented room | Yes | Yes | Yes | Parents and siblings | Social grant |
| 8 | VRM8 | Mangaung | Formal settlement | 2021/07/16 |  | MUCPP | Vaginal | 39 | 2700 g | Unknown | No | Exclusive breastfeeding |  |  | 4 | 7,0 | 18,7 | 26,3 | House | Yes | Yes | Yes | Mother and family members | Social grant |
| 9 | VRM9 | Mangaung | Formal settlement | 2021/07/14 | F | Pelonomi | Vaginal | 39 | 3310 g | Yes | No | Exclusive breastfeeding |  |  | 6 | 7,3 | 19,0 | 26,6 | House | Yes | Yes | Yes | Parents | Social grant |
| 10 | VRM10 | Mangaung | Formal settlement | 2021/07/29 | F | Pelonomi | Vaginal | 38 | 3300 g | No | No | Exclusive breastfeeding |  |  | 4 | 7,1 | 16,7 | 25,3 | House | Yes | Yes | Yes | Mother and family members | Social grant |
| 11 | VRM11 | Mangaung | Formal settlement | 2021/07/29 | M | Pelonomi | Vaginal | 39 | 2710 g | No | No | Exclusive breastfeeding |  |  | 4 | 7,1 | 16,7 | 24,4 | House | Yes | Yes | Yes | Parents | Social grant |
| 12 | VRM12 | Mangaung | Township | 2021/07/29 | F | Pelonomi | Vaginal | 40 | 3450 g | No | No | Exclusive breastfeeding |  |  | 4 | 7,1 | 16,9 | 25,3 | House | Yes | Yes | Yes | Mother and family members | Both |
| 13 | VRM13 | Mangaung | Formal settlement | 2021/08/12 | F | Pelonomi | Vaginal | 38 | 2810 g | Yes | No | Exclusive breastfeeding |  |  | 4 | 8,4 | 16,4 | 26,3 | House | Yes | Yes | Yes | Parents and siblings | Social grant |
| 14 | VRM14 | Mangaung | Formal settlement | 2021/08/20 | F | Pelonomi | Vaginal | 36 | 3330 g | No | No | Exclusive breastfeeding |  |  | 3 | 7,9 | 16,1 | 26,1 | House | Yes | Yes | Yes | Mother and family members | Social grant |
| 15 | VRM15 | Mangaung | Informal settlement | 2021/08/19 | F | Pelonomi | Vaginal | 40 | 2900 g | Yes | No | Exclusive breastfeeding |  |  | 4 | 8,0 | 16,4 | 25,3 | Shack | No | No | No | Parents and siblings | Social grant |
| 16 | VRM16 | Mangaung | Township | 2021/08/18 | F | Pelonomi | Vaginal | 38 | 3030 g | No | No | Exclusive breastfeeding |  |  | 6 | 8,0 | 20,3 | 26,4 | House | Yes | Yes | Yes | Mother and family members | Both |
| 17 | VRM17 | Mangaung | Formal settlement | 2021/08/19 | M | Pelonomi | Vaginal | 40 | 3950 g | Yes | No | Exclusive breastfeeding |  |  | 4 | 7,9 | 20,1 | 26,4 | Shack | Yes | No | Yes | Parents and siblings | Social grant |
